# Posthumous Works

**Published:** 1783

**Authors:** 


III *Œuvres pofthumes*, i. e. *Pofthumous Works of* M. Pouteau, M. D. *Surgeon in chief to the Hotel Dieu at Lyons.* 8vo. Paris, 1783. 3 vols.

THIS pofthumous collection is publifhed from M. Pouteau's manufcripts by M. Colombier, phyfician at Paris. Several of the effays it contains have already appeared in the author's *Melanges de chirurgie*, but are here reprinted with confiderable additions. Notes on particular paffages are given by the editor at the end of each volume.—We fhall confine our account chiefly to the new articles, as the *Melanges* are already well known in this country.

The firft volume begins with an inquiry into the nature of the cancerous virus, and the means of deftroying it. We here meet with a great deal of erroneous theory. Extirpation is very properly recommended as the only mode of cure, cure, and after this has been effected, we are advifed to apply the actual cautery to the wound that no part of the cancerous mafs may remain. Such a precaution, when the difeafe is within the reach of the knife, will to the Englifh furgeon perhaps appear cruel and unneceffary. The only remedy advifed after the extirpation is ice water given by itfelf, without any other nourifhment, for feveral weeks. This practice, we are told, was firft introduced by a capuchin in Malta, who from his fuccefs with this fimple medicine in vifceral and other obftructions, got the name of *medecin à l'eau fraiche.* M. Pouteau relates a cafe of fchirrous uterus cured by the fole ufe of this remedy. The patient drank five or fix bottles of ice water in the courfe of four and twenty hours, and on the eighth day a diarrhœa was brought on, which returned afterwards at intervals, and removed the complaint..

The effay on cancers is followed by fome obfervations on the abforbing pores of the fkin, and the operation of external remedies. The author offers feveral arguments (which we cannot admit) to prove that falivation is merely the effect of a fpafmodic irritation communicated to the the falival glands by the fkin or the inteftines through the medium of the nerves.

In a fupplement to a paper on the good effects of the actual cautery in rheumatifm, printed in the author's *Melanges*, he offers fome reflexions on a paffage in Suetonius, relative to the remedy employed by Auguftus againft the fciatica. His remarks on this fubject appear to be rather curious than ufeful. Thefe are followed by the cafe of a young lady who laboured under a painful difeafe of the hip joint, accompanied with hectic fever and night fweats. This complaint after refifting the hot baths of Bourbon l'Archambault, was cured by burning a cylinder of cotton on the part.

In the fourth memoir the author treats of pulmonary phthifis. A great number of cafes are related under the following heads, viz. *phlegmonic phthifis*; *eryfipelatous phthifis*; *phthifis from external caufes*; *fchirrous and cancerous phthifis*; *ferous phthifis*. Many of thefe accounts are tedious and uninterefting. The principal point the author endeavours to prove is the utility of blifters, and even of the actual cautery, in pulmonary cafes attended with an acute pain in the fide. He likewife mentions the good effects of of water-creffes in complaints of this kind. In the two cafes of what he calls fchirrous and cancerous phthifis, the affection of the lungs feems to have been merely fecondary. One of the patients had a cancer of the mouth, and the other a cancer of the breaft, but in neither of thofe cafes does there appear to have been a tranflation (as the author fuppofes) of the cancerous virus to the lungs.

The volume clofes with an effay on the rickets, in which blifters and the actual cautery are recommended as the principal means of cure.

The fecond volume begins with an effay on what the author calls "ferous and lymphatic fwellings of the joints, known by the name of falfe anchylofes." Here, as in the rickets, he relies chiefly on the actual cautery.

In the next paper M. Pouteau treats of the advantages and inconveniences of fire applied to the upper part of the head. It feems that an anonymous critic had charged him with having fuppreffed, in his *Melanges*, the cafe of an epileptic patient who fell a victim to this practice. Our author here endeavours to vindicate himfelf from this accufation. He firft quotes a paffage from De Haen, which exhibits the fenti-

ſentiments of a variety of writers from Hippocrates downwards, by whom this remedy is recommended, and then relates the hiſtory in queſtion. In this caſe, the epileptic ſymptoms having reſiſted a variety of remedies, M. Pouteau was induced to burn the bone at the upper part of the head with a hot iron. The patient died on the third day after the operation, and on diſſection a ſuppuration was diſcovered between the dura mater and the bone, and the whole of that membrane, as well as the pia mater, was in a ſtate of inflammation. Two caſes are quoted from De Haen's *Ratio Medendi*, in which a ſimilar operation proved equally fatal—M. Pouteau very candidly blames himſelf for having been ſilent "on a cataſtrophe which, "had it been rendered public in 1760, might "perhaps have reached the ear of M. De Haen, "and have ſaved the lives of two unfortunate "perſons."

In a paper on the means of recovering drowned perſons, M. Pouteau inſiſts much on the neceſſity of introducing a blow pipe through a wound made into the trachea, by means of which the moiſture in the bronchiæ may be drawn out, and freſh air thrown in. He likelikewise recommends, in such cases, the burning the soles of the feet with a hot iron.

We meet with a variety of judicious observations on fractures of the fore arm and fibula, which are the subjects of two essays. M. Pouteau is of opinion that a strong contraction of the pronator muscles may fracture the radius, and he has met with instances where a similar accident has happened to the fibula merely from a violent contraction of some of its muscles.

In an essay " on the appearance of life and " sensation, which may be excited in an ampu- " tated limb, and on animal entities (*entes ani-* " *males*)" the author offers some ingenious remarks on what he calls vegetative and vascular life, and which he supposes to be inherent in animal bodies, independently of the nerves and of what is stiled irritability. To this vegetative principle he attributes the re-union of parts that have been separated. He relates after Garengeot, the case that is likewise quoted by Van Swieten, of the nose that was bit off, and brought to unite again by proper care. He likewise mentions a case from Heister of a woman who having cut off the end of one of her fingers, had it replaced.

The

The firſt eſſay in the third volume relates to the phyſical cauſe of labour pains. In this paper he ſuppoſes the uterus to be compoſed of two ſorts of fibres, the one cellular, the other nervous. The latter, we are told, are poſſeſſed of extreme ſenſibility, and being incapable of diſtention beyond a certain degree, communicate the leaſt irritation they receive to the muſcular fibres, which immediately contract and preſs on the contents of the uterus. This theory is founded on a ſtructure of the uterus, which is altogether imaginary.

M. Pouteau next treats of the mechaniſm of parturition, and then preſents us with ſome obſervations on births that have happened after the uſual period, or what the French call *naiſſances tardives*. Two inſtances are related of delivery after the uſual term, one at the end of eleven months, and the other of eleven months and an half.

In a paper on the days of cutting for the ſtone (*ſur les jours de taille*). M. Pouteau offers ſome very judicious obſervations on an improper cuſtom which has but too long prevailed in the hoſpitals in France of performing that operation on a great number of patients the ſame day. In his *Melanges* he had very candidly acknow-

 ledged ledged that he never acted as a lithotomist twice in the same morning, without fearing that the second patient might have reason to complain of the labour and attention he had employed on the first. This passage, it seems, gave offence to the late M. le Cat, who fancied, and not without reason as our author acknowledges, that it alluded to him. M. le Cat, we are told, used every season to insert in different gazettes the number of patients he had cut for the stone on particular days, with the number of minutes employed on each. The account used to close with the total of hours and minutes in which this succession of operations had been accomplished, "as if (we use the words of M. Pou-"teau) the merit of a surgeon ought to be de-"termined by an hour glass." The author, in one part of his paper, compares days of this sort to an *auto da fé*.

Treating of gangrene, M. Pouteau relates three instances to prove that this affection is communicable by contact in the same manner as the plague and the small pox. These cases, however, in our opinion afford no ground for the conclusions he has ventured to draw from them. We shall content ourselves with mentioning one of them, the other two are of the same kind.

A young

A young man, who had venereal chancres, underwent the operation for a phymofis. The patient lodged in a healthy fituation, but his wound was dreffed with lint brought from the hofpital. The prepuce mortified, and our author attributes this circumftance to the lint which he fuppofes to have been impregnated with the virus of gangrene.

M. Pouteau condemns the ufe of the bark in mortifications, as he has always found it a very inefficacious remedy in fuch cafes. In its ftead he recommends camphor in large dofes, and externally the actual cautery or burning oil.

We are next prefented with fome reflections on the incontinence of urine, which fometimes remains after the operation for the ftone. Thefe remarks relate chiefly to the *lithotome caché*, an inftrument not ufed in this country.

In a fupplement are given fome obfervations, by the author's father, on the ufe of blifters in different difeafes.

An excellent engraved portrait of the author is prefixed to the firft volume.

